# Exploiting the similarity of dissimilarities for biomedical applications and enhanced machine learning

**DOI:** 10.1371/journal.pcbi.1012716

**Published:** 2025-01-24

**Authors:** Mohammad Neamul Kabir, Li Rong Wang, Wilson Wen Bin Goh

**Affiliations:** 1 Lee Kong Chian School of Medicine, Nanyang Technological University, Singapore, Singapore; 2 Center for Biomedical Informatics, Nanyang Technological University, Singapore, Singapore; 3 School of Computer Science and Engineering, Nanyang Technological University, Singapore, Singapore; 4 School of Biological Sciences, Nanyang Technological University, Singapore, Singapore; 5 Center of AI in Medicine, Nanyang Technological University, Singapore, Singapore; 6 Division of Neurology, Department of Brain Sciences, Faculty of Medicine, Imperial College London, London, United Kingdom; Origin Bioinformatics, CANADA

## Abstract

The “similarity of dissimilarities” is an emerging paradigm in biomedical science with significant implications for protein function prediction, machine learning (ML), and personalized medicine. In protein function prediction, recognizing dissimilarities alongside similarities provides a more detailed understanding of evolutionary processes, allowing for a deeper exploration of regions that influence biological functionality. For ML models, incorporating dissimilarity measures helps avoid misleading results caused by highly correlated or similar data, addressing confounding issues like the Doppelgänger Effect. This leads to more accurate insights and a stronger understanding of complex biological systems. In the realm of personalized AI and precision medicine, the importance of dissimilarities is paramount. Personalized AI builds local models for each sample by identifying a network of neighboring samples. However, if the neighboring samples are too similar, it becomes difficult to identify factors critical to disease onset for the individual, limiting the effectiveness of personalized interventions or treatments. This paper discusses the “similarity of dissimilarities” concept, using protein function prediction, ML, and personalized AI as key examples. Integrating this approach into an analysis allows for the design of better, more meaningful experiments and the development of smarter validation methods, ensuring that the models learn in a meaningful way.

## What is similarity?

When seeking to identify commonalities among items in a set, a logical approach is to focus on understanding the shared intrinsic characteristics of those items. This pursuit of similarity is intuitive and widely applied across various disciplines. In biomedical science, it plays a key role in revealing relationships between genes and organisms.

Many bioinformatics algorithms rely on similarity to draw new inferences and deepen our understanding of biological phenomena. The sequence alignment tool, BLAST [1], assesses the relationship between 2 sequences based on the extent of mutual similarity. By scrutinizing the extent of likeness between gene sequences, these algorithms help discern potential connections, ultimately contributing to the identification of functional and evolutionary relationships among genes [2–4]. Within bioinformatics, using similarity is effective in juxtaposing various biological entities, encompassing DNA, RNA, and protein sequences.

Analyzing protein sequence similarity is a powerful method for identifying homologous sequences [5–7]. It is expected that related proteins are characterized by a shared ancestral origin and exhibits resemblance in sequences, structures, and functions. It has been famously stated that “sequence determines structure,” [8,9]. This underscores the intricate relationship between the amino acid sequence in a protein and its resulting spatial arrangement, which ultimately determines its activity and role in living organisms. Thus, by leveraging protein sequence similarity, researchers can identify and categorize these homologous sequences, providing valuable insights into the evolutionary relationships and potential functional characteristics of proteins. For example, protein sequence similarity can be used to identify homologous sequences that share a common ancestry and are likely to have similar functions and structures [7]. Sequence similarity is also used to determine conserved and diverged regions of proteins and contributes towards identifying species-specific differences. Examples of such algorithms include BLAST [9], HomoloGene [10], BLAT [11], GeneWise [12], and FASTA [13].

By assessing similarities among sequences, we can deduce ancestral and speciation relationships, enabling the construction of comprehensive evolutionary trees and pathways [14]. Leveraging similarity in this manner allows us to formulate informed predictions and hypotheses regarding biological sequences. These insights serve as valuable guides for experimental studies, contributing to an enhanced comprehension of the intricate relationships among genes, proteins, and other molecules within living systems. The utilization of similarity as a tool in these analyses is pivotal for unraveling the complexities inherent in biological processes.

## What are the limitations of similarity?

While similarity is a valuable concept, its effective application is equally important. When comparing 2 highly similar objects, the potential for uncovering novel or useful information decreases. Moreover, if the comparison centers on irrelevant traits or features, it may lead to false assumptions about their relationship. Therefore, selecting appropriate features and applying a nuanced understanding of the context in which similarity is used are crucial for gaining meaningful insights.

Four key issues must be considered:

***1. The choice of comparisons***. During sequence comparison, when a match is obtained in spite of long evolutionary distance, this match is more likely to point towards the existence of functional significance. However, when matches are obtained without sufficient variation or evolutionary conservation, we are less assured that these are functionally significant. Alignments and similarity scores derived from selective comparisons is useful for uncovering overarching biological principles. For example, the presence of a Walker A motif is indicative of nucleotide-binding proteins (in some cases signaling protein) [15], while the presence of a serine protease catalytic triad is indicative of proteases [16]. However, the successful identification of these principles also depends on using the right data, e.g., using sequence sets with sufficient variations.
***2. Unstable representation and measurement of similarity due to biological complexity and wrong assumptions in parameterization***. The challenge of representing and measuring similarity in biological contexts stems from the inherent complexity of biological systems and the risk of errors in parameterization. In biology, comparing seemingly dissimilar objects is common due to factors like insertions, deletions, and duplications, which can cause significant variation in sequence length and complexity, even among related sequences. In practice, accurately aligning longer, more complex sequences become increasingly difficult, as small differences in alignment can lead to large discrepancies in similarity scores. Addressing sequence length variations often requires introducing gaps or breaks to properly align the sequences [17]. As a result, the complexity of biological entities and careful selection of algorithm parameters are critical to avoiding potential pitfalls, such as false-positive or false-negative predictions.
***3. Similarity may not capture meaningful relationships when divergence is the main driver***. The limitation of relying solely on similarity arises when evolutionary divergence plays a pivotal role. Throughout evolutionary processes, various events such as gene duplication, deletion, or divergence can occur, which give rise to homologous sequences with significant divergence, resulting in low similarity scores, despite retaining functional or structural similarities. On the other hand, non-homologous sequences might exhibit similar sequences due to convergent evolution or random chance, introducing the risk of false-positive predictions of similarity. Moreover, a nuanced scenario involves convergent evolution leading to non-homologous sequences with dissimilar sequences that nonetheless perform similar functions. Unfortunately, algorithms that solely focus on superficial similarity may overlook such instances. In essence, understanding meaningful relationships in biological sequences requires considering the intricate interplay of evolutionary processes and discerning between divergence-driven differences and instances of convergent evolution that produce functionally similar outcomes.
***4. Sequence similarity is dependent on the reliability*, *coverage*, *and completion of existing knowledge***. The effectiveness of sequence similarity in predicting protein function hinges on the reliability, coverage, and completeness of existing knowledge, as highlighted in our previous work on protein function prediction [18]. Protein function prediction algorithms commonly rely on the assumption that proteins within the same family should exhibit significant similarity. While it is reasonable to expect similarity among proteins of the same functional family, the reality is that some protein members deviate significantly in similarity from others. This variability poses a challenge for methods that depend solely on similarity measures. Despite the inherent diversity in similarity levels among family members, most protein function prediction algorithms assess their performance using data sets containing both highly similar and low-similarity proteins. However, the inclusion of low-similarity proteins in the test set is typically limited, and they represent a minority. Consequently, the overall performance metrics of these algorithms do not truly reflect their ability to predict the function of proteins with lower similarity. The consequence is an inflated performance evaluation that may not accurately gauge the algorithm’s efficacy in handling the diversity inherent in protein families.

### Measures of dissimilarity and identifying the “similarity of dissimilarities”

Although intuitive, similarities are often used to identify shared characteristics; however, these shared traits may not always be reflected in obvious ways. For example, in genomics, a researcher studying genes involved in cell cycle regulation might initially focus on common sequence motifs or structural features to understand their functions. Yet, not all genes involved in this process will necessarily exhibit similar sequences or structures, despite contributing to the same regulatory pathway. Therefore, a thorough analysis must go beyond surface-level similarities and consider the functional aspects that link the genes. In such cases, incorporating dissimilarity measures alongside similarity assessments can be useful.

Dissimilarity measures identify shared distinctions that may not be evident through traditional similarity measures. This approach enables a more comprehensive understanding of relationships within and across groups, emphasizing the conservation of dissimilarities as a key factor in grouping objects based on their unique characteristics. In cases where the similarity signal is not strong enough, dissimilarity measures help to enhance those weak signals and makes the mapping easier for such difficult proteins.

Dissimilarity measures or differences between signals have been used to find patterns in signal processing [19–21], improving efficiency and costs. In data mining applications: the Minkowski distance (or *l*p-*norm*) is an example of a well-known dissimilarity measure [22]. Dissimilarities are also used in text mining to detect outliers from a collection of text (also known as novelty detection, anomaly detection, deviation detection, etc.) [23]. Dissimilarities are relatively underexplored in the realms of biomedical and biological science. Hence, this paper aims to delve into the significance of incorporating dissimilarity measures, shedding light on why this concept is crucial and identifying specific areas within these scientific domains where the consideration of dissimilarities can yield valuable insights.

### How gene function prediction algorithms fail because they have not seen enough “meaningfully diverse” examples

**Fig 1:**
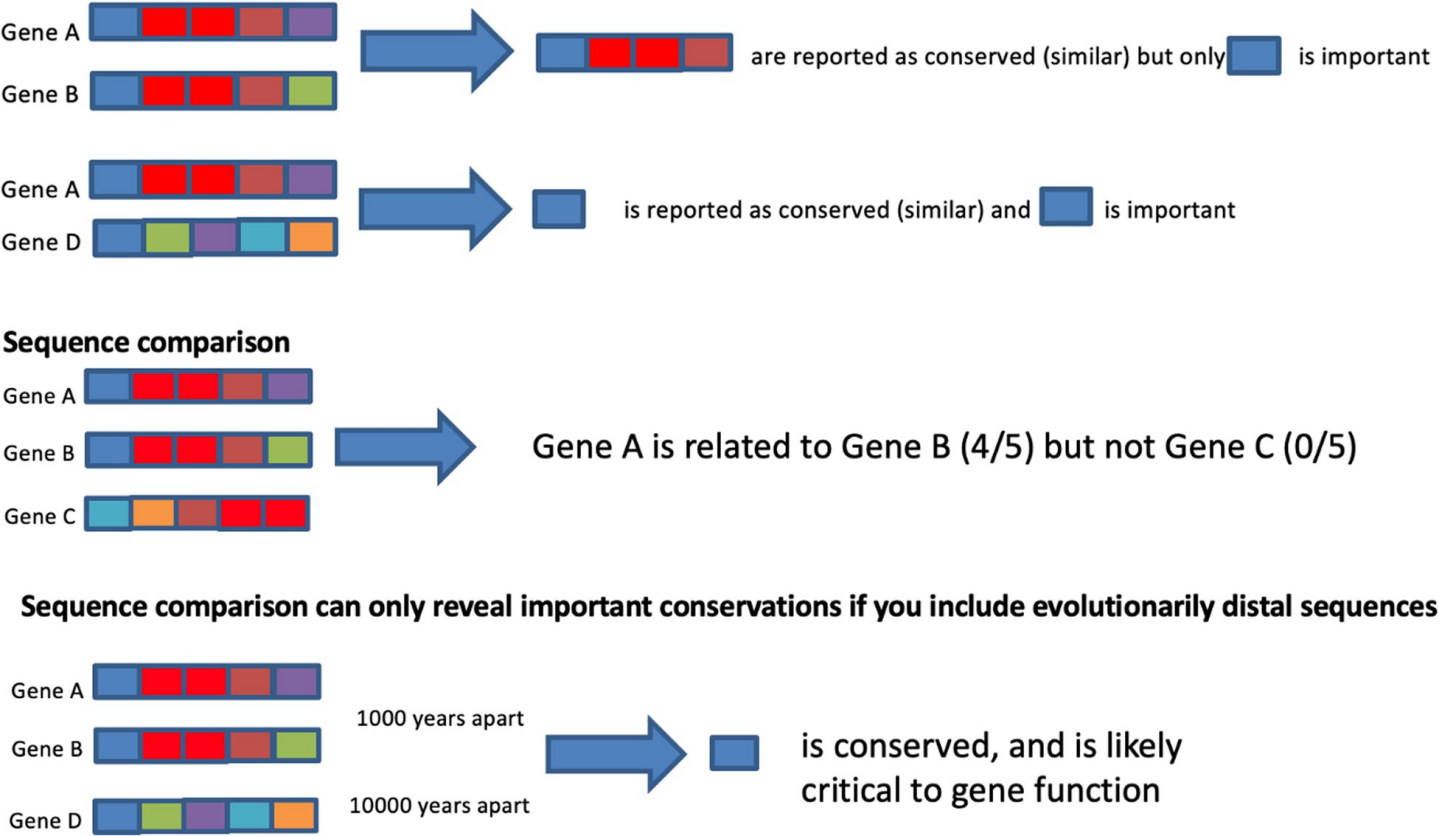
Similarity can reveal useful meaning if you compare with the right things. Gene A is being compared against 2 other genes, B and C. By direct comparison, Gene A would be deemed as related to B but not C. Gene A is highly similar to Gene B, and thus, produces a generous block of conserved regions. However, if we were to compare with gene D, which is evolutionarily more distant, then only the blue motif is revealed as conserved, and more likely critical to gene function. However, note that with gene C and gene D, there is limited similarity to A.

Similarity can reveal useful insight if we compare the right things. To illustrate this with a toy example (**Fig 1)**, we compare Gene A against 2 other genes, B and C.

Via direct comparison of sequence similarities, Gene A would be deemed as related to B but not C. Since Gene A is similar to Gene B, we observe a generous block of conserved regions (4 out of 5). However, if we were to compare with a new sequence, Gene D (1 out of 5), which is evolutionarily more distant, then only the blue motif is revealed as conserved, and likely critical to gene function. However, note that with Genes C and D, both have limited similarity to A. This makes it difficult to differentiate cases of low similarity with conserved function, and of low similarity without conserved function.

We can get around this issue by exploiting prior knowledge. Suppose we are aware that Genes A, B, and D have conserved function, we may elect to compare only these sequences and identify the minimum regions that are conserved. The relations among these sequences can also be expressed using simple tools, such as a dendrogram. The key is to ensure we include sufficiently diverse examples as “positive” examples for our algorithm so that we may identify important regions that are functionally conserved.

In the next step, we can apply contrastive learning via comparisons against “negative” examples. Identified functionally conserved regions can be contrasted with protein sequence families of known different functions that may include Gene C and other sequences that could be superficially similar. By identifying what is minimally conserved among examples of the same function and ensuring these conservations do not occur in examples of different function, we increase the confidence of finding something useful and not just non-causally correlated/associated.

### The model validation step in machine learning requires meaningful variation

Earlier, we see that the availability of diversity helps us to choose the correct evolutionarily conserved regions that are important for function. The statistical procedure of selecting these conserved regions is known as feature selection. Feature selection is useful for promoting model explainability if selected features are relevant and non-substitutable. It is performed using statistical learning techniques like correlation analysis, mutual information, or extracting feature importance scores. It can also be assayed via meta-analysis techniques, and methods such as divergent and convergent validation [24,25].

**Fig 2:**
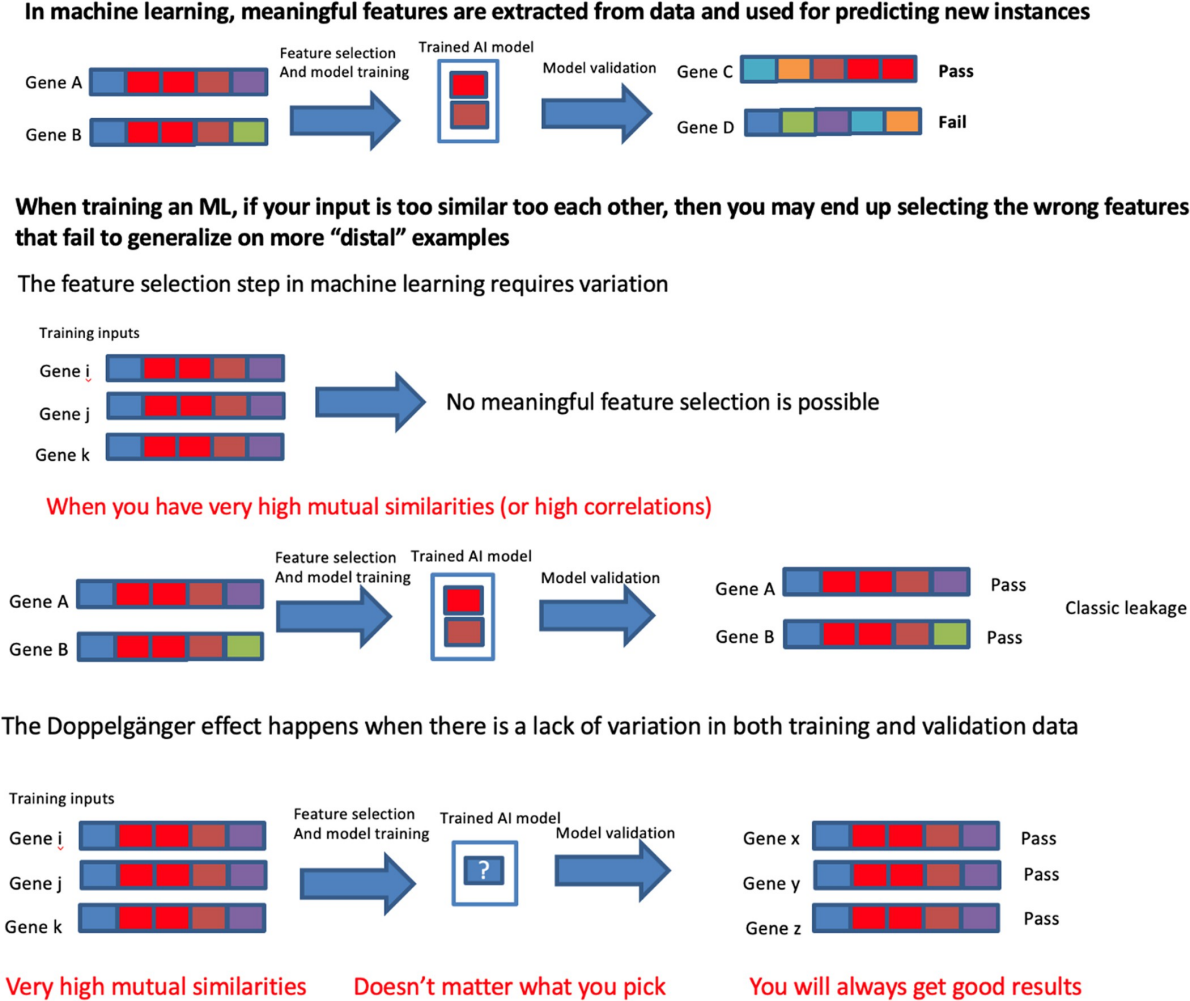
Having sufficient variation in training data is important.

In our next example (**Fig 2**), we return to Genes A and B again. Recall in **Fig 1**, Genes A and B shared 4/5 regions and were considered highly similar. A separate comparison with Gene D which also shared similar function revealed that only the blue region is critical for functional conservation, rendering the red and brown regions as non-causally associated.

If the input is too similar, we may select wrong features. In **Fig 2**, the red and brown regions were mistakenly selected. And so, the trained model fails to correctly predict Gene D, which also shares the same function. And depending on how the information was used, this trained model may also predict Gene C as sharing the same function, which has a red region albeit in a different (wrong) position.

In situations where training samples exhibit high similarity, the choice of selected features becomes inconsequential, as any classifier trained on such data consistently yields favorable results on the test set (**Fig 2**). Traditional techniques like cross-validation are ineffective in mitigating this issue, as the consistently positive outcomes persist. This phenomenon, identified as the Doppelgänger Effect, occurs when highly correlated or similar samples consistently yield favorable outcomes in AI/ML training [26,27].

Next, we discuss how the “similarity of dissimilarities” can be applied in 3 areas: enhanced protein function prediction via EnsembleFam, resolving Doppelgänger effects, and in a form of AI/ML known as Transductive Personalized learning (TPL).

### EnsembleFam

While building protein annotation methods, sequence similarity (and other forms of information that reflect homology) among members of the same protein family plays an important role. Most methods try to benefit from this information and build a good model to infer new members. While many proteins within the same family share similarity, a significant portion that only shares limited similarity or has very weak homological relation with other members exist. These proteins are more difficult to annotate compared to the high similarity ones. Most existing methods do not perform well on such proteins where the similarity is limited. This is because most methods to date rely heavily on sequence similarity information to infer function.

For proteins with limited similarity, its difference against other proteins is useful. Almost all existing methods disregard dissimilarity information to annotate proteins. However, we devised a novel strategy (EnsembleFam) to build a predictive model relying highly on dissimilarity features [18].

**Fig 3:**
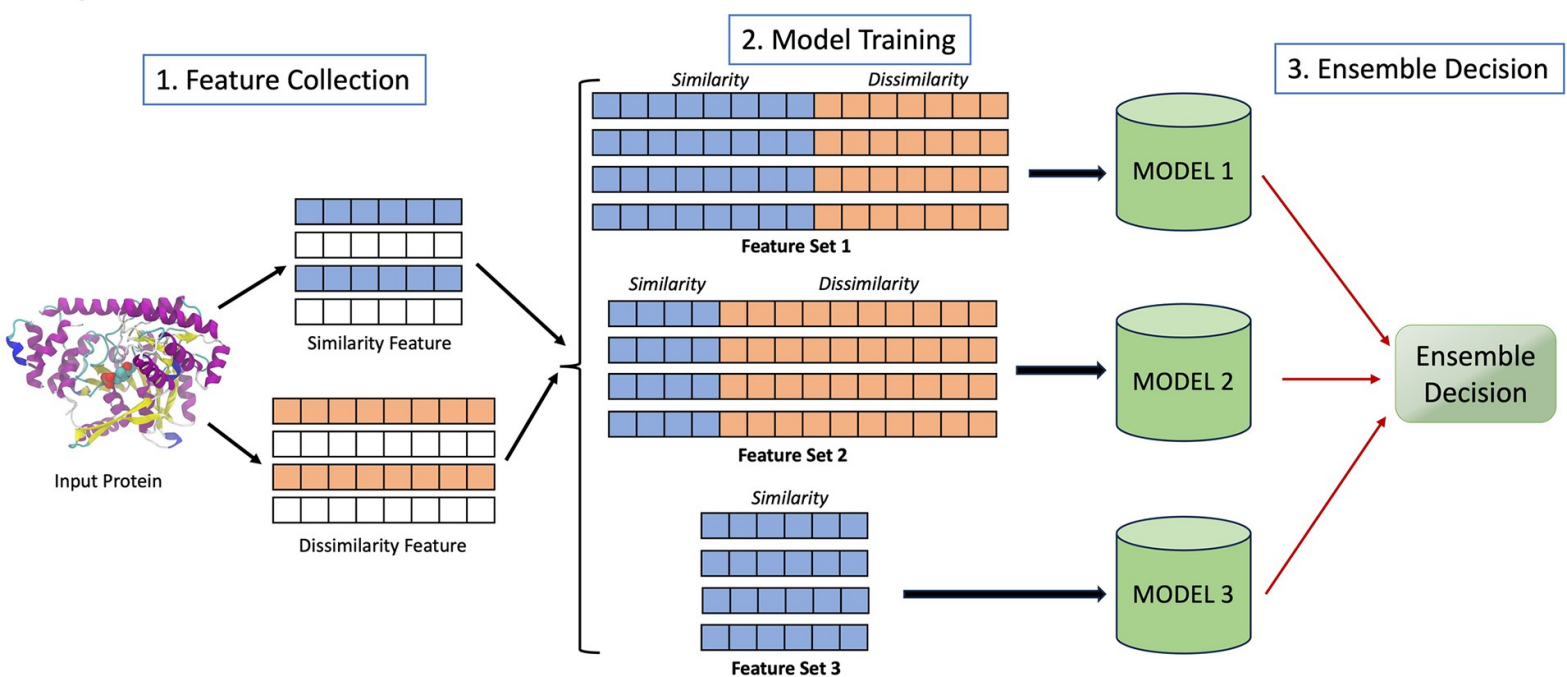
EnsembleFam model architecture in 3 steps: feature collection, model training, and ensemble decision. For each protein family, a model is built to identify its members using a combination of similarity and dissimilarity features.

EnsembleFam is an ML approach based on training SVM models using combination of similarity and dissimilarity features (**Fig 3)**. To represent a protein sequence, similarity scores are collected from proteins of the same class while dissimilarity scores are collected based on comparisons against proteins from other classes. Thus, a class-wise similarity-dissimilarity feature vector is generated for any given sequence and later used to identify the function of that protein. Most existing methods only use similarity scores. This limits the method in only being able to predict easy proteins (having higher similarity with the reference proteins) in the test set. As shown in EnsembleFam [18], if we segregate the test set into easy and difficult proteins (based on similarity with reference), most methods fail to provide accurate prediction for the difficult twilight zone proteins. In contrast, EnsembleFam [18] benefits from dissimilarity features and provides consistent performance for both easy and difficult proteins.

Extensive experiments were conducted to compare EnsembleFam [18] with other methods using COG data set [28] and GPCR data set [29], which depicts the power of dissimilarity features in the context of protein function prediction. A snapshot of the results on the twilight zone proteins is demonstrated in Table 1. The detailed result can be found in the original manuscript of EnsembleFam [18]. Although the overall performance for all the methods in both data sets is over 95% on the whole test set, the performance drops drastically in the twilight zone as shown in Table 1. EnsembleFam performs better overall leveraging on the dissimilarity features.

**Table 1:** Performance comparison of EnsembleFam with other methods on the twilight zone (identity < = 30%) of COG and GPCR data sets. In COG data set, the results are divided into 6 subgroups based on number of predictions made by EnsembleFam, where EnsembleFam and pHMM provides more than 1 prediction (indicated by predCount) and DeepFam provides exactly one. If one of the predictions in a subgroup is correct for EnsembleFam and pHMM, and the only one for DeepFam is correct, then it is assumed as correct. All the results displayed here are average of 5-fold cross-validation.

COG data set						
Method	predCount = 1	predCount = 2	predCount = 3	predCount = 4	predCount = 5	predCount > 5
EnsembleFam	**72.07**	**81.00**	**82.82**	**84.96**	**85.33**	**85.27**
pHMM	69.54	73.75	55.51	70.62	70.85	73.55
DeepFam	57.14	54.52	49.90	46.92	43.64	35.94
GPCR data set						
Method	Sub-subfamily		Sub-family		Family	
EnsembleFam	**30.92**		**45.15**		**65.45**	
pHMM	5.51		11.76		39.80	
DeepFam	5.53		16.88		61.44	

### Doppelgänger effects

**Fig 4:**
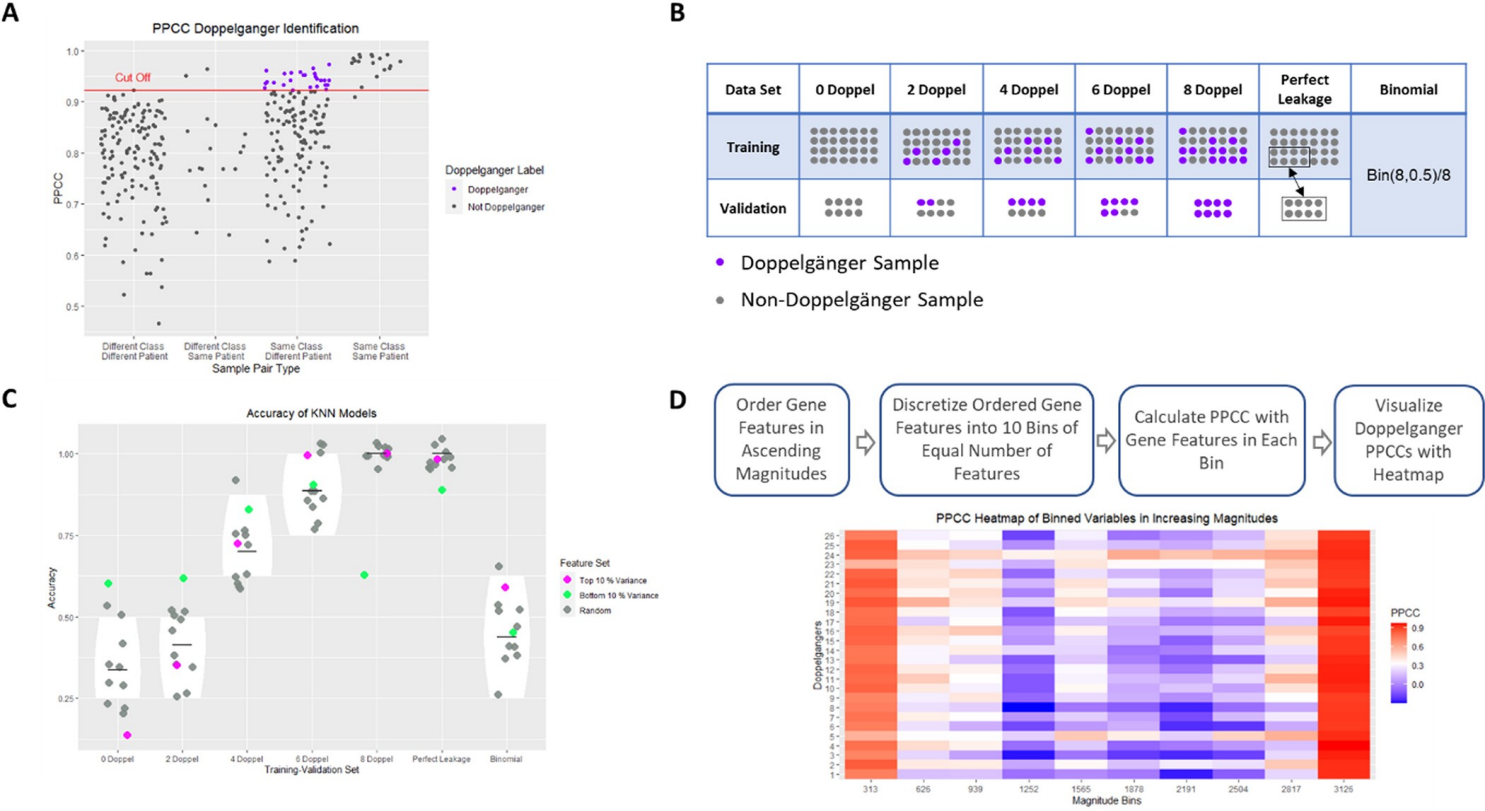
Depictions of Data Doppelganger Identification (DDI) (Panel A), the experiment setup for Doppelgänger Inflation Test (DIT) (Panel B), the expected “leakage-like” effect during DIT (Panel C) and the procedure of identifying highly correlated features.

The Doppelgänger effect is a form of pseudo-leakage due to high mutual correlations across biological samples. Samples with high mutual correlations as known as Data Doppelgängers. When enriched in data can lead to high performing random ML models. To know whether data is plagued by high presence of Data Doppelgängers, we may apply 2 procedures: a check on the distribution of inter-sample pairwise similarities, stratified across class and patient labels (**Fig 4A**) and an estimation on the inflationary effect of Data Doppelgängers when split across training and validation data in a procedure known as the Doppelgänger Inflation Test (DIT) (**Fig 4B**). In DIT, we carefully assort samples into the training and validation sets to form training-validation subsets with increasing proportions of Data Doppelgängers. ML models with randomly selected feature sets are then trained and evaluated on each of these data. The greater the improvements in model performance as the proportion of Data Doppelgängers increase, the stronger their inflationary effects.

Suppose we learn that there is high presence of Data Doppelgängers, such that we observe high similarity distributions and a clear “leakage-like” effect during DIT (**Fig 4C**). Before interpreting the trained model, we should ask whether our collected data is representative of the population [30], and if so, whether such high similarity distributions are expected. If the data is not representative of the population (verifiable through statistical testing), then the researcher should review their data sampling protocols and resume their data collection efforts.

When high similarity is expected, all samples will appear very similar regardless of whether more data is collected. To deal with this, we may identify regions of high similarities and mask them from further analysis, focusing instead on the remaining regions where variation is observed. This approach is a deployment of the concept of “similarity of dissimilarities” into action.

In our example, the goal is to build a predictive model for predicting renal cell carcinoma in a proteomics data set consisting of 36 samples with an equal number of tumor and normal samples. Each sample has 3,126 features. Previous research [27] has identified 26 Data Doppelgängers in this data set. We segmented the features into bins, which act as new features (**Fig 4D**). Given the considerable number of features, binning is necessary for effective visualization of feature correlations. We first ordered the features by their magnitudes (the sum of feature) before partitioning into 10 bins of equal frequency (number of gene features). We calculate the pairwise similarities across samples for every bin using Pearson Pairwise Correlation Coefficient (PPCC) to identify which bins are strongly associated with Doppelgänger Effects. These bins are then removed from further analysis.

Some masked bins contain meaningful information. If more structured approaches are required, we can test for specificity by testing if the features are predictive of completely unrelated phenotypes [31]. We can apply convergence validation to isolate the most supported subset of features, followed by divergence validation to determine if the identified features work on all validation test and can beat random signatures [24,32,33]. To understand how random and irrelevant gene signatures can predict breast cancer prognosis where most genes are disrupted (and therefore associated with the cancer), this set of approaches allowed us to isolate a subset of proliferation genes (the Super Proliferation Set; SPS) as critical, and which are directly involved in the good performance of many published clinical biomarkers.

### Transductive personalized learning

Transductive Personalized Learning (TPL) is a form of AI/ML that constructs personalized models for each sample based on the set of “most similar” neighboring samples [34–36]. TPL is often employed to develop personalized models for diseases, allowing for tailored therapeutic approaches [35]. It has shown promise in the classification of lymphoma and modeling of EEG signals [35,37]. By estimating the value of the function at a given point rather than attempting to approximating a global function, TPL is theoretically robust against heterogeneity as it avoids over-generalization.

The success of TPL is dependent on whether neighboring samples are meaningfully distributed. Using the double weighted k Nearest Neighbors (WWKNN) as a simple example, we may encounter the following issue(s). In WWKNN, the k-nearest of a sample are taken to be most pertinent to itself where k is usually set as the square root of the number of samples [38]. Suppose we have a sample size of 81, then the value of k would be √81 = 9. However, it is not guaranteed that all samples will have at least 9 samples in its neighborhood (may end up drawing irrelevant samples into the local model). Conversely, even if there are 9 samples in the vicinity, it does not mean all 9 contribute useful signal. Some of them could be technical or biological replicates contributing bias if not identified and resolved. Hence, TPL work on the premise that local neighborhood samples are “similar” to the sample under consideration, and therefore more relevant and can give context-specific insights, is important for yielding insight.

Hence, if dissimilarities are not adequately considered, the personalized models may not accurately capture the variabilities present in the disease specific to an individual in terms unique starting points in disease development, disease progression/etiology, and formulating therapies specific to individual or subtypes of diseases.

TPL is thus, relevant to the exploration of dissimilarities in the following ways:

***Minimizing Heterogeneity***: TPL aims to minimize heterogeneity among samples by identifying personalized features (e.g., molecular signatures or biomarkers) for each sample. However, if the neighboring samples are doppelgängers of each other or with the sample under consideration, non-meaningful signal inflation can occur. This situation fails to inform on the idiosyncratic causal factors pertinent to each personalized model.

***Handling Dissimilarities***: A challenge arises when neighboring samples exhibit unusually strong dissimilarities or divergences (possibly, due to a one or few outliers due to wrong definition of local neighborhood). Traditional TPL models might struggle in scenarios where such strong dissimilarities are more pronounced. In such cases, dissimilarity measures need to be appropriately handled to prevent non-meaningful signal inflation and ensure accurate personalized models.

***Capturing Subpopulation Effects***: TPL, by defining a neighborhood per sample, operates in a manner that captures subpopulation effects. Identifying unique dissimilarities across subpopulations are important for understanding unique starting points in disease development and identifying specific targets relevant to individuals or disease subtypes. Identifying common dissimilarities among subpopulations are important for understanding convergence points in disease development.

The relevance of TPL towards the similarity of dissimilarities lies in the ability to build meaningful personalized models while navigating challenges posed by dissimilarities among samples. Addressing dissimilarities appropriately is essential for preventing non-meaningful signal inflation and enhancing the accuracy and reliability of personalized models in biomedical applications.

## Conclusions

The concept of the “similarity of dissimilarities” is important for advancing biomedical science. From protein function prediction to machine learning and artificial intelligence, it has broad applicability and implications. Understanding how to harness dissimilarities can lead towards robust applications and meaningful biomedical insights.
